# Evaluation of the diagnostic accuracy of a new point-of-care rapid test for SARS-CoV-2 virus detection

**DOI:** 10.1186/s12967-020-02651-y

**Published:** 2020-12-21

**Authors:** Leonardo Miscio, Antonio Olivieri, Francesco Labonia, Gianfranco De Feo, Paolo Chiodini, Giuseppe Portella, Luigi Atripaldi, Roberto Parrella, Rodolfo Conenna, Franco Maria Buonaguro, Ernesta Cavalcanti, Paolo Ascierto, Gerardo Botti, Attilio Bianchi

**Affiliations:** 1 Istituto Nazionale Tumori Di Napoli, IRCCS “G. Pascale”, Naples, Italy; 2 Gaboservice, Caserta, Italy; 3 Università Degli Studi Della Campania Luigi Vanvitelli, Naples, Italy; 4 Azienda Ospedaliera Universitaria “Federico II”, Naples, Italy; 5 Azienda Ospedaliera dei Colli – PO Cotugno, Naples, Italy

**Keywords:** SARS-CoV-2, COVID-19, Point-of-care rapid test, Health surveillance

## Abstract

**Background:**

The easy access to a quick diagnosis of coronavirus disease 2019 (COVID-19) is a key point to improve the management of severe acute respiratory syndrome coronavirus 2 (SARS-CoV-2) and to contain its spread. Up to now, laboratory real-time PCR is the standard of care, but requires a fully equipped laboratory and significant infrastructure. Consequently, new diagnostic tools are required.

**Methods:**

In the present work, the diagnostic accuracy of the point-of-care rapid test "bKIT Virus Finder COVID-19" (Hyris ^Ltd^) is evaluated by a retrospective and a prospective analysis on SARS CoV-2 samples previously assessed with an FDA “authorized for the emergency use—EUA” reference method. Descriptive statistics were used for the present study.

**Results:**

Results obtained with the Hyris Kit are the same as that of standard laboratory-based real time PCR methods for all the analyzed samples. In addition, the Hyris Kit provides the test results in less than 2 h, a significantly shorter time compared to the reference methods, without the need of a fully equipped laboratory.

**Conclusions:**

To conclude, the Hyris kit represents a promising tool to improve the health surveillance and to increase the capacity of SARS-CoV-2 testing.

## Background

The infection from severe acute respiratory syndrome coronavirus 2 (SARS-CoV-2), and the resulting coronavirus disease 2019 (COVID-19) present important diagnostic challenges [1, 2]. In particular, improved access to diagnostics is a key point to counteract the spread of the virus [3]. Different diagnostic strategies are now available to identify or exclude a current infection, identify people in need of care escalation, or to test for past infection and immune response [4, 5].

Laboratory real-time (RT)-PCR represents the standard of care for the detection of the SARS-CoV-2 infection [6, 7], but this technique is time-consuming (up to 24 h for the result), not always available, and the technical requirements usually can only be met by centralized diagnostic laboratories.

Point-of-care diagnostics tests to detect current SARS-CoV-2 infection have the potential to allow an earlier detection of infection, compared to laboratory-based diagnostic methods, thus contributing to the reduction of household and community transmission [8–10]. A rapid diagnosis is also crucial for setting up a good therapy [11, 12]. In addition, they can be operated in near-patient settings rather than in the laboratory, which are expected to be more easily accessible and to relieve laboratory workload. For this reason, these kinds of rapid tests were identified by a WHO expert group as one of the priorities in response to the COVID-19 outbreak [13].

The aim of the present study is to evaluate the diagnostic accuracy of the point-of-care rapid test "bKIT Virus Finder COVID-19" produced by Hyris ^Ltd^ (hereafter, termed Hyris Kit) for the detection of the SARS-CoV-2 virus, against the used reference method (SARS-CoV-2 RNA research by the RT-PCR method). The Hyris Kit is designed to be easy and quick to use in emergency condition or in delocalized areas; thus, this work is intended also to ensure that the time required to run the test, providing the same sensitivity and specificity, is less than the reference method.

## Material and methods

### Study design and sample collection

The diagnostic accuracy of the Hyris Kit was evaluated by a retrospective and a prospective analysis on SARS-CoV-2 samples previously assessed (and validated positive and negative) by an FDA “authorized for the emergency use—EUA” reference method. The study was conducted in the Department of Laboratory Medicine of the National Institute for The Study and Treatment of Cancer IRCCS "Fondazione G Pascale" (IRCCS Pascale) of Naples (Italy).

For the retrospective analysis, SARS CoV-2-positive nasopharyngeal swab samples were taken by during the pandemic peak period (from March to June 2020) and were provided by the Azienda Ospedaliera Universitaria Federico II (Federico II University Hospital) and the Azienda Ospedaliera dei Colli—Ospedale Domenico Cotugno (Cotugno Hospital) of Naples (Italy). SARS-CoV-2-negative nasopharyngeal swab samples were collected in July 2020 by the IRCCS Pascale.

For the prospective analysis, new positive samples were prepared by Federico II University Hospital starting from pools of one or two positive samples, diluted in a negative sample (pooled samples).

All participant patients were aged  ≥ 18 years; they understood and signed the informed consent form. The study protocol was approved by the Ethics Committee of IRCCS Pascale.

### Reference analysis methods

The reference methods, used to verify the positivity or negativity of collected samples for the retrospective analysis were: (1) Abbott Kit: *Real-Time SARS-CoV-2 (#09N77-095)*, EUA by the FDA, where a complete analysis run lasts for 6 h on average, which detect the RdRP and the N gene of the SARS-CoV-2; and (2) Roche Kit: *LightMix®Modular SARS-Cov-2 (COVID19) RdRP*, where a complete analysis run lasts for 3 h on average, which detects the RdRP and the E gene of the SARS-CoV-2. Using the Roche Kit, samples are rated negative if they have a late amplification beyond 35 threshold cycle (Ct, Kit cut-off) or no amplification for the target gene.

Positive samples by the Federico II University Hospital and negative samples by IRCCS Pascale were analyzed with the Abbott Kit following the manufacturer’s instructions. Positive samples by Cotugno Hospital were analyzed with the Roche Kit following the manufacturer’s instructions.

The Roche Kit, which needs 200 μL of sample, was used in place of the Abbott Kit if the sample volume was not enough for the specifications of the Abbott Kit, which requires 800 μL. The prospective analysis on pooled samples was first carried out with the Hyris Kit, and then with the Abbott kit.

All samples were analyzed in compatible soil (universal transport media/viral transport medium) and then stored at − 80 °C.

### Hyris Kit point-of-care test

**Fig. 1:**
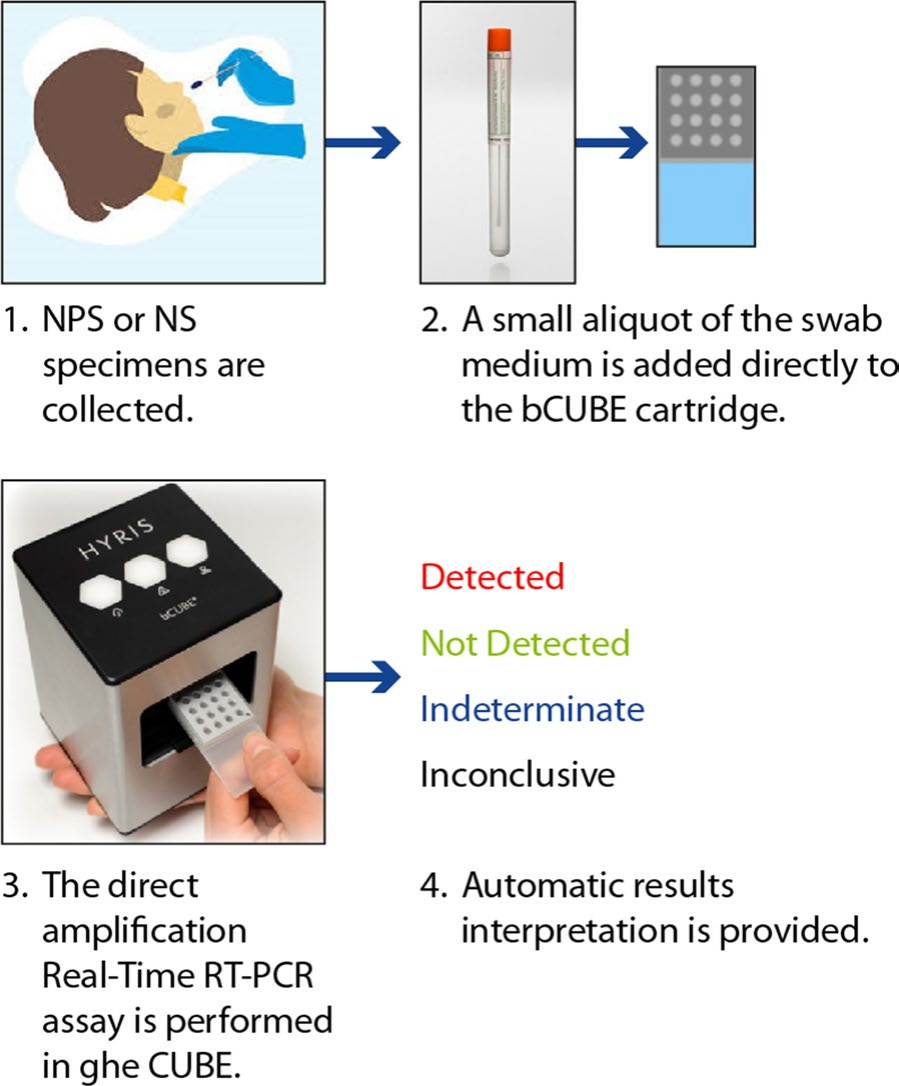
Hyris Kit schematic of the workflow

The Hyris Kit is intended for use on the bCUBE instrument, a miniaturized real-time PCR for the in vitro qualitative detection of SARS-CoV-2 nucleic acid in nasopharyngeal swabs or nasal swabs specimens (Fig. 1). No sample extraction is needed prior to the PCR amplification step. The Hyris Kit contains reagents that allow the sample direct amplification in the analysis well. These reagents are designed to reduce the impact of the RNase contained into the clinical specimen, as well as to promote the nucleic acid extraction before the retro transcriptase step. To allow a quick detection of the disease, without necessarily going through an analysis laboratory, all the solutions required for the analysis process are premixed within the kit. After the nasopharyngeal or nasal swab collection, a small aliquot of the swab transport medium (5 μL), which contains the specimen, can be directly added to 15 μL of the reaction mix, previously loaded into the bCUBE cartridge well (16 samples on each standard cartridge, 36 samples cartridge will be soon available). The sample will be amplified as per the amplification kit procedure. During the RT step of the RT-PCR thermal protocol, the viral RNA will be released into the qPCR master mix. The direct amplification RT-PCR assay is performed in the bCUBE. The bCUBE is designed to be fully portable and to work in network and the embedded automatic results interpretation delivers a test output readable by non-experts. At the end of the analysis, a “detected” or “not detected” result can be assigned to each sample for positive and negative samples, respectively. The Hyris Kit automatic interpretation algorithm classifies all those samples that do not show amplification for viral targets and very late amplification for endogenous control, namely the human gene *RPP30* (i.e., the ubiquitous gene that encodes for RNase P), as "*indeterminate*". Late amplification of the *RPP30* gene may depend on a poorly performed sample, this can alter the outcome of the analysis in the case of a patient with a viral load very close to the limit of the detection of the method, causing cases of "false negativity". Therefore, for the present study, samples with an "*indeterminate*" outcome are excluded from the trial.

Samples analyzed with the Hyris Kit that have the amplification of only one of the two targets of the SARS-CoV-2 virus are classified as "*inconclusive*". Samples with "*inconclusive*" results are excluded from the trial for the present study.

In case of positive samples that showed a no amplification signal or showed partial amplification during retrospective analysis with the Hyris kit, analyses were repeat with the Roche Kit.

### Determination of the lower limit of detection of the Hyris kit

Two positive samples provided by the Federico II, analyzed with the Hyris Kit and evaluated positive, were diluted and tested in technical duplication in order to identify a concentration of analyte beyond the limit of detection of the method of analysis. All dilutions where one or more replicated were not properly amplified (both targets for both replicated) have been discarded. Having defined the dilution to be tested, the samples were processed with the Roche Kit to identify the correlation between the Ct obtained with Hyris Kit, and the Roche Kit, in order to exclude all samples that have Ct values greater than the threshold value identified, which corresponds to 33 Ct.

### Blinded analysis

To perform a blinded analysis with Hyris kit, a selection of positive and negative samples was prepared and by IRCCS Pascale staff and provided anonymously for analysis to Hyris kit staff, who carried out the analysis. At the end of the tests, IRCCS Pascale's staff disclosed all the information for data analysis.

### Statistical analysis

Descriptive statistics were used for the present study.

## Results

### Study samples

For the retrospective analysis, a total of 37 positive samples were provided by the Federico II University Hospital. A total of the 206 negative samples were provided by IRCCS Pascale.

A total of 20 positive samples were provided by Cotugno Hospital and were used for the blind trial test. Baseline characteristics of the relative patients are summarized in Table 1.

**Table 1:** Baseline characteristics of patients

Characteristics	Federico II University Hospital (positive samples)	Cotugno Hospital (positive samples)	IRCCS Pascale (negative samples)
Males, n (%)	16 (43)	5 (25)	91 (45)
Age, mean (SD)	46 (19)	37 (19)	50 (17)
Symptomatology: n (%)			
Symptomatic	5 (14)	9 (45)	
Asymptomatic	16 (43)	7 (35)	
Pauci-symptomatic	16 (43)	4 (20)	

For the prospective analysis, 30 new positive samples were provided by the Federico II University Hospital, by using the pool method.

### Evaluation of Hyris Kit diagnostic accuracy

#### Retrospective analysis

Of 37 positive samples provided by Federico II, three samples gave “*inconclusive*” results with Hyris kit, meaning one in two viral targets was not amplified correctly. In total, 11 samples gave no amplification signals with the Hyris kit. These 14 samples that failed the Hyris Kit test were retested with Roche Kit, as it was assumed that the samples could be degraded due to repeated thawing, the time and the conditions of storage. Analysis with Roche Kit confirmed that these 14 samples had failed, so they were not considered in this study.

The remaining 23 positive samples provided by Federico II were tested positive by the Hyris Kit (see Additional file 1: Table S1 for details of the results).

Considering the negative samples, 203 were classified as negative with the Hyris Kit. Three samples, while being negative for virus markers, were interpreted by the Hyris Kit automatic interpretation algorithm as "*indeterminate*", consequently were not considered in the present study (see Additional file 1: Table S2 for more details).

#### Prospective analysis

All 30 positive samples obtained from pools of different samples resulted positive with the first Hyris Kit test and subsequent Abbott Kit test, performed to confirm the result (see Additional file 1: Table S3 for details).

#### Blind trial test

Of the 20 positive samples provided by Cotugno Hospital, four samples were excluded before the start of the trial because they met one or more of the exclusion criteria.

Consequently, in the blind trial 60 samples were analyzed: 16 positive samples provided by the Cotugno Hospital, 14 positive pool samples and 30 negative samples.

Of the 16 positive samples tested from the Cotugno Hospital, six were excluded. In particular, two positive samples were excluded from the trial due to Ct greater than the determined threshold value (33 Ct), two samples because of negative results, and two because they resulted as “*inconclusive*” with the Hyris Kit.

The results obtained in the tests conducted with Hyris Kit on the remaining 54 samples are equivalent to those obtained with the reference methods (see Additional file 1: Table S4 for more details).

## Discussion

Even if current medical knowledge on the management of COVID-19 patients and experimental treatments are still evolving, different protocols to minimize the risk of infection among the general population [14], patients and healthcare workers have been approved and diffused by International Health Authorities [15]. In this context, the development of point-of-care rapid tests to consent an earlier detection of SARS-CoV-2 infection is an urgent need, to allow an effective and rapid plan of diagnosing and to prevent further transmission [16].

This study was designed to evaluate the diagnostic accuracy of the point-of-care rapid test Hyris Kit.

In total, 63 positive samples and 203 negative samples were included in the study. Results show that the point-of-care Hyris Kit provides the same results of standard laboratory-based RT-PCR methods for all the analyzed samples. Observing the comparative results (see Additional file 1), the Ct are different among the different methods of analysis. The several reasons that explain these data includes: the absence of the purification of nucleic acids step for the Hyris kit (direct amplification); the different genes analyzed by the different methods, which are expressed at different stages of the pathology; the possible process of degradation that the samples may have encountered during the period between the analysis performed with the reference method and the test conducted with Hyris kit.

Of note, the Hyris Kit allows to get the results in less than 2 h, much quicker than the reference methods evaluated, maintaining the same sensitivity and specificity.

Therefore, the Hyris Kit diagnostic accuracy has been confirmed, along with its advantage in terms of time to results.

Another key advantage of this point-of-care platform is that it is a fully automated direct sample-to-answer platform, removing the need for the laboratory infrastructure required for traditional RT-PCR.

Therefore, this method can be used also in all medical or pediatricians’ offices since the availability of an equipped laboratory or other technological tools is no longer required.

Consequently, the results of the present study have a considerable socio-economic impact. Indeed, the Hyris Kit represents a method to promote a quick and easy health surveillance in all contexts in which a fully equipped laboratory is not accessible and rapid results are required (i.e., cruise ships, flights, patients to be hospitalized, sports teams) and responds to the request for the development of to use point-of-care assays, due to the urgent clinical and public health need to increase SARS-CoV-2 testing capacity.

For instance, the Hyris Kit has been recently approved as a point-of-care test in Canada for human diagnostics as an Interim Order.

## Conclusion

The COVID-19 pandemic requires the set-up of solid and reliable diagnostic tests in order to ease the management of the pandemic spreading and its consequences. This work demonstrates the diagnostic accuracy of the point-of-care rapid test "bKIT Virus Finder COVID-19" (Hyris ^Ltd^), sustaining the use of this promising tool to improve the health surveillance and to increase SARS-CoV-2 testing capacity.

## Supplementary Information

**Additional file 1: Table S1. **Results obtained by analyzing the 37 positive samples provided by Federico II University Hospital and their inclusion or exclusion from the trial. **Table S2. **Results obtained by analyzing the 206 negative samples included in the trial, provided by IRCCS Pascale and their inclusion or exclusion from the trial. **Table S3. **Results obtained by analyzing the 30 pooled samples prepared by Federico II University Hospital and their inclusion or exclusion from the trial. **Table S4.** Results obtained by the blind analysis of the 60 samples and their inclusion or exclusion included from the trial.

## Data Availability

The datasets used and/or analyzed during the current study are available from the corresponding author on reasonable request.

## Abbreviations

SARS-CoV-2: Severe acute respiratory syndrome coronavirus 2
COVID-19: Coronavirus disease 2019
RT: Real-time
Hyris Kit: BKIT Virus Finder COVID-19
Ct: Threshold cycle
