# Supplementary material for: Evaluation of the diagnostic accuracy of a new point-of-care rapid test for SARS-CoV-2 virus detection
Source: J Transl Med. 2020 Dec 21;18:488. doi: 10.1186/s12967-020-02651-y (PMC7752099; doi:10.1186/s12967-020-02651-y)
Supplement: Supplementary file 1 — Additional file 1: Table S1. Results obtained by analyzing the 37 positive samples provided by Federico II University Hospital and their inclusion or exclusion from the trial. Table S2. Results obtained by analyzing the 206 negative samples included in the trial, provided by IRCCS Pascale and their inclusion or exclusion from the trial. Table S3. Results obtained by analyzing the 30 pooled samples prepared by Federico II University Hospital and their inclusion or exclusion from the trial. Table S4. Results obtained by the blind analysis of the 60 samples and their inclusion or exclusion included from the trial. [file 12967_2020_2651_MOESM1_ESM.docx]

**SUPPLEMENTARY MATERIAL**

**Table S1: Results obtained by analyzing the 37 positive samples provided by Federico II University Hospital and their inclusion or exclusion from the trial.**

| **ID Trial** | **Sample ID** | **Ct Abbott Kit** | **Ct Roche Kit** | **Ct Hyris Kit Mix 1** | **Ct Hyris Kit Mix 2** | **Ct Hyris Kit RP** | **Abbott Kit or Roche Kit result** | **Hyris Kit result** | **Inclusion in the trial** |
| --- | --- | --- | --- | --- | --- | --- | --- | --- | --- |
| 91 | 95107019050 | 17 |  | 33,37 | 33,89 | 29,36 | Positive | Positive | Included |
| 92 | 95107019048 | 27 | 39.20 | N/A | N/A | 30,91 | Negative | Negative | Excluded |
| 93 | 2 | 30 | 39.43 | N/A | N/A | 34,34 | Negative | Negative | Excluded |
| 97 | 95107019263 | 17 |  | 32,35 | 33,25 | 30 | Positive | Positive | Included |
| 98 | 95107139245 | 20 |  | 35,6 | 35,37 | 29,98 | Positive | Positive | Included |
| 99 | 3 | 15 |  | 21,65 | 21,62 | 26,22 | Positive | Positive | Included |
| 103 | 95107139217 | 28 | N/A | N/A | N/A | 30,91 | Negative | Negative | Excluded |
| 104 | 95107139215 | 21 |  | 38,03 | 39,85 | 30,8 | Positive | Positive | Included |
| 105 | 4 | 27 |  | 34,56 | 34,48 | 29,69 | Positive | Positive | Included |
| 109 | 95107089157 | 17 |  | 34,42 | 35,99 | 29,46 | Positive | Positive | Included |
| 110 | 95107089155 | 24 | N/A | N/A | N/A | 30,82 | Negative | Negative | Excluded |
| 111 | 5 | 28 |  | 37,1 | 36,13 | 33,18 | Positive | Positive | Included |
| 115 | 95104287460 | 8 |  | 28,13 | 28,87 | 29,06 | Positive | Positive | Included |
| 116 | 1 | 20 | 37.74 | 38,14 | N/A | 31,4 | Negative | Indeterminate | Excluded |
| 117 | 6 | 23 |  | 39,59 | 38,7 | 30,08 | Positive | Positive | Included |
| 121 | 7 | 34 | 38.72 | N/A | N/A | 32,39 | Negative | Negative | Excluded |
| 122 | 8 | 15 |  | 22,3 | 23,4 | 31,92 | Positive | Positive | Included |
| 123 | 9 | 28 |  | 29,94 | 30,55 | 29,78 | Positive | Positive | Included |
| 127 | 10 | 25 |  | 30,19 | 39,04 | 29,35 | Positive | Positive | Included |
| 128 | 11 | 20 |  | 36,7 | 37,92 | 29,04 | Positive | Positive | Included |
| 129 | 12 | 14 |  | 35,1 | 35,31 | 30,27 | Positive | Positive | Included |
| 133 | 13 | 22 | 36.18 | 38,98 | N/A | 28,72 | Negative | Indeterminate | Excluded |
| 134 | 14 | 24 |  | 38,95 | 38,84 | 29,79 | Positive | Positive | Included |
| 135 | 15 | 23 | N/A | N/A | N/A | 28,53 | Negative | Negative | Excluded |
| 139 | 16 | 24 | N/A | N/A | N/A | 28,15 | Negative | Negative | Excluded |
| 140 | 17 | 17 |  | 22,32 | 22,45 | 29,22 | Positive | Positive | Included |
| 141 | 18 | 20 | N/A | N/A | N/A | 27,4 | Negative | Negative | Excluded |
| 145 | 19 | 20 |  | 32,89 | 33,72 | 26,68 | Positive | Positive | Included |
| 146 | 20 | 16 |  | 35,83 | 37,23 | 28,63 | Positive | Positive | Included |
| 147 | 21 | 21 | N/A | N/A | N/A | 29,1 | Negative | Negative | Excluded |
| 153 | 22 | 35 | N/A | N/A | N/A | 33,36 | Negative | Negative | Excluded |
| 157 | 23 | 32 |  | 36,43 | 38,96 | 34,5 | Positive | Positive | Included |
| 158 | 24 | 23 | N/A | 39,5 | N/A | 29,5 | Negative | Indeterminate | Excluded |
| 160 | 25 | 18 |  | 36,34 | 38,09 | 31,26 | Positive | Positive | Included |
| 164 | 26 | 22 |  | 38,93 | 39,89 | 29,75 | Positive | Positive | Included |
| 165 | 27 | 29 |  | 34,02 | 35,82 | 28,43 | Positive | Positive | Included |
| 166 | 28 | 36 | N/A | N/A | N/A | 32,58 | Negative | Negative | Excluded |

**Table S2: Results obtained by analyzing the 206 negative samples included in the trial, provided by IRCCS Pascale and their inclusion or exclusion from the trial.**

| **ID Trial** | **Sample ID** | **Ct Hyris Kit RP** | **Abbott Kit result** | **Hyris Kit result** | **Inclusion in the trial** |
| --- | --- | --- | --- | --- | --- |
| 1 | 208875 | 29,32 | Negative | Negative | Included |
| 2 | 208871 | 31,59 | Negative | Negative | Included |
| 3 | 208852 | 28,91 | Negative | Negative | Included |
| 4 | 208863 | 32,99 | Negative | Negative | Included |
| 5 | 208894 | 29,99 | Negative | Negative | Included |
| 6 | 208902 | 31,65 | Negative | Negative | Included |
| 7 | 208890 | 29,62 | Negative | Negative | Included |
| 8 | 208855 | 31,76 | Negative | Negative | Included |
| 9 | 208854 | 27,86 | Negative | Negative | Included |
| 10 | 208873 | 28,47 | Negative | Negative | Included |
| 11 | 208882 | 31,00 | Negative | Negative | Included |
| 12 | 208857 | 28,32 | Negative | Negative | Included |
| 13 | 208895 | 31,76 | Negative | Negative | Included |
| 14 | 208920 | 29,78 | Negative | Negative | Included |
| 15 | 208887 | 28,97 | Negative | Negative | Included |
| 16 | 208884 | 30,46 | Negative | Negative | Included |
| 17 | 208892 | 30,77 | Negative | Negative | Included |
| 18 | 208853 | 29,57 | Negative | Negative | Included |
| 19 | 208867 | 31,45 | Negative | Negative | Included |
| 20 | 208874 | 31,42 | Negative | Negative | Included |
| 21 | 208880 | 32,14 | Negative | Negative | Included |
| 22 | 208851 | 32,02 | Negative | Negative | Included |
| 23 | 208848 | 31,50 | Negative | Negative | Included |
| 24 | 208849 | 27,35 | Negative | Negative | Included |
| 25 | 208868 | 30,68 | Negative | Negative | Included |
| 26 | 208878 | 31,18 | Negative | Negative | Included |
| 27 | 208915 | 28,83 | Negative | Negative | Included |
| 28 | 208847 | 26,43 | Negative | Negative | Included |
| 29 | 208846 | 28,56 | Negative | Negative | Included |
| 30 | 208866 | 28,08 | Negative | Negative | Included |
| 31 | 208860 | 27,38 | Negative | Negative | Included |
| 32 | 208893 | 29,15 | Negative | Negative | Included |
| 33 | 208897 | 29,58 | Negative | Negative | Included |
| 34 | 208889 | 27,52 | Negative | Negative | Included |
| 35 | 208858 | 28,57 | Negative | Negative | Included |
| 36 | 208914 | 30,34 | Negative | Negative | Included |
| 37 | 208869 | 29,01 | Negative | Negative | Included |
| 38 | 208865 | 29,75 | Negative | Negative | Included |
| 39 | 208850 | 29,96 | Negative | Negative | Included |
| 40 | 208856 | 30,68 | Negative | Negative | Included |
| 41 | 208870 | 26,35 | Negative | Negative | Included |
| 42 | 208896 | 32,39 | Negative | Negative | Included |
| 43 | 208876 | 30,89 | Negative | Negative | Included |
| 44 | 208891 | 29,98 | Negative | Negative | Included |
| 45 | 208901 | 28,66 | Negative | Negative | Included |
| 46 | 208861 | 30,12 | Negative | Negative | Included |
| 47 | 208877 | 28,99 | Negative | Negative | Included |
| 48 | 208879 | 29,11 | Negative | Negative | Included |
| 49 | 208918 | 28,84 | Negative | Negative | Included |
| 50 | 208905 | 29,47 | Negative | Negative | Included |
| 51 | 208673 | 36,51 | Negative | Indeterminate | Excluded |
| 52 | 208653 | 29,50 | Negative | Negative | Included |
| 53 | 208669 | 29,51 | Negative | Negative | Included |
| 54 | 208672 | 26,57 | Negative | Negative | Included |
| 55 | 208664 | 32,55 | Negative | Negative | Included |
| 56 | 208677 | 31,27 | Negative | Negative | Included |
| 57 | 208668 | 28,45 | Negative | Negative | Included |
| 58 | 208670 | 30,43 | Negative | Negative | Included |
| 59 | 208667 | 28,77 | Negative | Negative | Included |
| 60 | 208674 | 28,23 | Negative | Negative | Included |
| 61 | 208676 | 30,70 | Negative | Negative | Included |
| 62 | 208665 | 32,59 | Negative | Negative | Included |
| 63 | 208900 | 30,05 | Negative | Negative | Included |
| 64 | 208675 | 30,14 | Negative | Negative | Included |
| 65 | 208666 | 29,89 | Negative | Negative | Included |
| 66 | 208678 | 28,52 | Negative | Negative | Included |
| 67 | 208671 | 30,17 | Negative | Negative | Included |
| 68 | 208687 | 32,40 | Negative | Negative | Included |
| 69 | 208725 | 30,39 | Negative | Negative | Included |
| 70 | 208721 | 32,12 | Negative | Negative | Included |
| 71 | 208719 | 32,37 | Negative | Negative | Included |
| 72 | 208722 | 29,31 | Negative | Negative | Included |
| 73 | 208718 | 29,72 | Negative | Negative | Included |
| 74 | 208714 | 27,70 | Negative | Negative | Included |
| 75 | 208716 | 31,34 | Negative | Negative | Included |
| 76 | 208685 | 30,94 | Negative | Negative | Included |
| 77 | 208710 | 29,53 | Negative | Negative | Included |
| 78 | 208713 | 29,99 | Negative | Negative | Included |
| 79 | 208705 | 31,51 | Negative | Negative | Included |
| 80 | 208712 | 29,11 | Negative | Negative | Included |
| 81 | 208696 | 28,88 | Negative | Negative | Included |
| 82 | 208701 | 27,94 | Negative | Negative | Included |
| 83 | 208720 | 28,21 | Negative | Negative | Included |
| 84 | 208694 | 28,95 | Negative | Negative | Included |
| 85 | 208700 | 31,94 | Negative | Negative | Included |
| 86 | 208723 | 30,98 | Negative | Negative | Included |
| 87 | 208707 | 25,22 | Negative | Negative | Included |
| 88 | 208706 | 29,63 | Negative | Negative | Included |
| 89 | 208691 | 29,33 | Negative | Negative | Included |
| 90 | 208692 | 28,29 | Negative | Negative | Included |
| 94 | 208697 | 30,23 | Negative | Negative | Included |
| 95 | 208724 | 28,87 | Negative | Negative | Included |
| 96 | 208699 | 29,61 | Negative | Negative | Included |
| 100 | 208709 | 29,93 | Negative | Negative | Included |
| 101 | 208688 | 30,77 | Negative | Negative | Included |
| 102 | 208704 | 34,39 | Negative | Negative | Included |
| 106 | 208654 | 29,77 | Negative | Negative | Included |
| 107 | 208686 | 28,11 | Negative | Negative | Included |
| 108 | 208695 | 31,51 | Negative | Negative | Included |
| 112 | 208782 | 31,38 | Negative | Negative | Included |
| 113 | 208993 | 30,83 | Negative | Negative | Included |
| 114 | 208802 | 30,74 | Negative | Negative | Included |
| 118 | 208831 | 31,64 | Negative | Negative | Included |
| 119 | 208749 | 30,00 | Negative | Negative | Included |
| 120 | 208784 | 29,79 | Negative | Negative | Included |
| 124 | 208755 | 30,34 | Negative | Negative | Included |
| 125 | 208768 | 28,19 | Negative | Negative | Included |
| 126 | 208763 | 30,74 | Negative | Negative | Included |
| 130 | 208764 | 30,15 | Negative | Negative | Included |
| 131 | 208747 | 29,51 | Negative | Negative | Included |
| 132 | 208773 | 29,23 | Negative | Negative | Included |
| 136 | 208760 | 30,26 | Negative | Negative | Included |
| 137 | 208779 | 29,86 | Negative | Negative | Included |
| 138 | 208761 | 30,38 | Negative | Negative | Included |
| 142 | 208752 | 28,94 | Negative | Negative | Included |
| 143 | 208756 | 30,34 | Negative | Negative | Included |
| 144 | 208754 | 33,86 | Negative | Negative | Included |
| 148 | 208770 | 28,70 | Negative | Negative | Included |
| 149 | 208777 | 28,18 | Negative | Negative | Included |
| 150 | 208780 | 30,21 | Negative | Negative | Included |
| 154 | 208762 | 31,55 | Negative | Negative | Included |
| 155 | 208798 | 28,94 | Negative | Negative | Included |
| 156 | 208751 | 32,20 | Negative | Negative | Included |
| 161 | 208702 | 31,75 | Negative | Negative | Included |
| 162 | 208774 | 29,32 | Negative | Negative | Included |
| 163 | 208783 | 31,89 | Negative | Negative | Included |
| 167 | 208776 | 31,80 | Negative | Negative | Included |
| 168 | 208748 | 27,76 | Negative | Negative | Included |
| 169 | 208758 | 27,93 | Negative | Negative | Included |
| 173 | 208703 | 34,02 | Negative | Negative | Included |
| 174 | 208772 | 32,50 | Negative | Negative | Included |
| 175 | 208753 | 29,81 | Negative | Negative | Included |
| 176 | 208789 | 32,51 | Negative | Negative | Included |
| 177 | 208800 | 31,53 | Negative | Negative | Included |
| 178 | 208805 | 31,90 | Negative | Negative | Included |
| 179 | 208799 | 31,45 | Negative | Negative | Included |
| 180 | 208801 | 30,61 | Negative | Negative | Included |
| 181 | 208810 | 31,72 | Negative | Negative | Included |
| 182 | 208829 | 30,87 | Negative | Negative | Included |
| 183 | 208842 | 32,68 | Negative | Negative | Included |
| 184 | 208793 | 32,26 | Negative | Negative | Included |
| 185 | 208806 | 29,64 | Negative | Negative | Included |
| 186 | 208792 | 27,5 | Negative | Negative | Included |
| 187 | 208796 | 31,19 | Negative | Negative | Included |
| 188 | 208790 | 38,18 | Negative | Indeterminate | Excluded |
| 189 | 208788 | 31,19 | Negative | Negative | Included |
| 190 | 208786 | 31,44 | Negative | Negative | Included |
| 191 | 208819 | 31,14 | Negative | Negative | Included |
| 192 | 208794 | 29,81 | Negative | Negative | Included |
| 193 | 208795 | 30,74 | Negative | Negative | Included |
| 194 | 208785 | 30,46 | Negative | Negative | Included |
| 195 | 208811 | 29,77 | Negative | Negative | Included |
| 196 | 208835 | 32,09 | Negative | Negative | Included |
| 197 | 208816 | 32,37 | Negative | Negative | Included |
| 198 | 208812 | 29,55 | Negative | Negative | Included |
| 199 | 208807 | 31,08 | Negative | Negative | Included |
| 200 | 208808 | 29,32 | Negative | Negative | Included |
| 201 | 208815 | 32,44 | Negative | Negative | Included |
| 202 | 208809 | 27,68 | Negative | Negative | Included |
| 203 | 208813 | 30,98 | Negative | Negative | Included |
| 204 | 208818 | 30,2 | Negative | Negative | Included |
| 205 | 208833 | 28,91 | Negative | Negative | Included |
| 206 | 208823 | 29,67 | Negative | Negative | Included |
| 207 | 208836 | 30,33 | Negative | Negative | Included |
| 208 | 208820 | 30,45 | Negative | Negative | Included |
| 209 | 208821 | 31,83 | Negative | Negative | Included |
| 210 | 208803 | 29,83 | Negative | Negative | Included |
| 211 | 208826 | 30,13 | Negative | Negative | Included |
| 212 | 208759 | 33,1 | Negative | Negative | Included |
| 213 | 208817 | 28,27 | Negative | Negative | Included |
| 214 | 208838 | 31,88 | Negative | Negative | Included |
| 215 | 208825 | 31,39 | Negative | Negative | Included |
| 216 | 208757 | 29,47 | Negative | Negative | Included |
| 217 | 208840 | 32,63 | Negative | Negative | Included |
| 218 | 208837 | 31,83 | Negative | Negative | Included |
| 219 | 208839 | 30,59 | Negative | Negative | Included |
| 220 | 208841 | 30,89 | Negative | Negative | Included |
| 221 | 208822 | 31,82 | Negative | Negative | Included |
| 222 | 208834 | 31,46 | Negative | Negative | Included |
| 223 | 208830 | 30,24 | Negative | Negative | Included |
| 224 | 208097 | 29,26 | Negative | Negative | Included |
| 225 | 208099 | 30,26 | Negative | Negative | Included |
| 226 | 208098 | 32,52 | Negative | Negative | Included |
| 227 | 208093 | 30,09 | Negative | Negative | Included |
| 228 | 208094 | 29,08 | Negative | Negative | Included |
| 229 | 208104 | 29,25 | Negative | Negative | Included |
| 230 | 208086 | 30,74 | Negative | Negative | Included |
| 231 | 208071 | 26,5 | Negative | Negative | Included |
| 232 | 208105 | 29,64 | Negative | Negative | Included |
| 233 | 208081 | 29,5 | Negative | Negative | Included |
| 234 | 208004 | 32,17 | Negative | Negative | Included |
| 235 | 208903 | 36,96 | Negative | Indeterminate | Excluded |
| 236 | 208781 | 30,18 | Negative | Negative | Included |
| 237 | 208766 | 28,91 | Negative | Negative | Included |
| 238 | 208771 | 30,81 | Negative | Negative | Included |
| 239 | 208750 | 30,6 | Negative | Negative | Included |
| 240 | 208765 | 32,45 | Negative | Negative | Included |
| 241 | 208767 | 29,43 | Negative | Negative | Included |
| 242 | 208909 | 29,94 | Negative | Negative | Included |
| 243 | 208913 | 29,95 | Negative | Negative | Included |
| 244 | 208917 | 29,96 | Negative | Negative | Included |
| 245 | 208904 | 28,5 | Negative | Negative | Included |
| 246 | 208907 | 27,31 | Negative | Negative | Included |
| 247 | 208908 | 29,83 | Negative | Negative | Included |
| 249 | 208899 | 28,94 | Negative | Negative | Included |
| 250 | 208916 | 32,97 | Negative | Negative | Included |

**Table S3: Results obtained by analyzing the 30 pooled samples prepared by Federico II University Hospital and their inclusion or exclusion from the trial.** The results were confirmed by the Abbott reference test.

| **ID pool sample** | **ID Trial** | **Original sample ID** | **Original sample result** | **Ct Abbott Kit** | **Ct Hyris Kit Mix 1** | **Ct Hyris Kit Mix 2** | **Ct Hyris Kit RP** | **Abbott Kit result** | **Hyris Kit result** | **Inclusion in the trial** |
| --- | --- | --- | --- | --- | --- | --- | --- | --- | --- | --- |
| 122245 | 245 | 208904 | Negative | 12,55 | 29,39 | 29,83 | 28,93 | Positive | Positive | Included |
|  | 122 | 8 | Positive |  |  |  |  |  |  |  |
| 122164248 | 248 | 208898 | Negative | 12,84 | 29,23 | 29,85 | 30,52 | Positive | Positive | Included |
|  | 164 | 26 | Positive |  |  |  |  |  |  |  |
|  | 122 | 8 | Positive |  |  |  |  |  |  |  |
| 122104249 | 104 | 95107139215 | Positive | 12,35 | 28,91 | 29,48 | 29,91 | Positive | Positive | Included |
|  | 249 | 208906 | Negative |  |  |  |  |  |  |  |
|  | 122 | 8 | Positive |  |  |  |  |  |  |  |
| 122219 | 219 | 208839 | Negative | 12,62 | 29,53 | 29,98 | 30,85 | Positive | Positive | Included |
|  | 122 | 8 | Positive |  |  |  |  |  |  |  |
| 122247 | 247 | 208908 | Negative | 12,68 | 29,1 | 29,41 | 29,84 | Positive | Positive | Included |
|  | 122 | 8 | Positive |  |  |  |  |  |  |  |
| 99212 | 212 | 208759 | Negative | 12,63 | 28,6 | 28,74 | 32,64 | Positive | Positive | Included |
|  | 99 | 3 | Positive |  |  |  |  |  |  |  |
| 9924 | 24 | 208849 | Negative | 12,55 | 30,56 | 30,8 | 27,42 | Positive | Positive | Included |
|  | 99 | 3 | Positive |  |  |  |  |  |  |  |
| 9912729 | 29 | 208846 | Negative | 13,04 | 29,15 | 29,76 | 29,14 | Positive | Positive | Included |
|  | 127 | 10 | Positive |  |  |  |  |  |  |  |
|  | 99 | 3 | Positive |  |  |  |  |  |  |  |
| 9912723 | 127 | 10 | Positive | 13,86 | 28,41 | 28,98 | 30,79 | Positive | Positive | Included |
|  | 23 | 208848 | Negative |  |  |  |  |  |  |  |
|  | 99 | 3 | Positive |  |  |  |  |  |  |  |
| 9916418 | 164 | 26 | Positive | 14,08 | 29,32 | 29,79 | 29,41 | Positive | Positive | Included |
|  | 18 | 208853 | Negative |  |  |  |  |  |  |  |
|  | 99 | 3 | Positive |  |  |  |  |  |  |  |
| 140225 | 225 | 209099 | Negative | 13,80 | 31,39 | 31,49 | 30,44 | Positive | Positive | Included |
|  | 140 | 17 | Positive |  |  |  |  |  |  |  |
| 14022 | 22 | 208851 | Negative | 13,60 | 30,12 | 30,71 | 33,19 | Positive | Positive | Included |
|  | 140 | 17 | Positive |  |  |  |  |  |  |  |
| 14021 | 21 | 208880 | Negative | 19,05 | 32,48 | 32,57 | 29,62 | Positive | Positive | Included |
|  | 140 | 17 | Positive |  |  |  |  |  |  |  |
| 1409817 | 160 | 25 | Positive | 15,13 | 34,2 | 34,98 | 27,82 | Positive | Positive | Included |
|  | 17 | 208892 | Negative |  |  |  |  |  |  |  |
|  | 140 | 17 | Positive |  |  |  |  |  |  |  |
| 1409816 | 98 | 95107139245 | Positive | 15,23 | 36,04 | 37,66 | 29,88 | Positive | Positive | Included |
|  | 16 | 208884 | Negative |  |  |  |  |  |  |  |
|  | 140 | 17 | Positive |  |  |  |  |  |  |  |
| 11520 | 20 | 208874 | Negative | 17,65 | 38,2 | 36,81 | 29,54 | Positive | Positive | Included |
|  | 115 | 95104287460 | Positive |  |  |  |  |  |  |  |
| 11515 | 15 | 208887 | Negative | 20,33 | 36,85 | 37,9 | 30,24 | Positive | Positive | Included |
|  | 115 | 95104287460 | Positive |  |  |  |  |  |  |  |
| 1159815 | 98 | 95107139245 | Positive | 19,74 | 34,54 | 35,59 | 32,21 | Positive | Positive | Included |
|  | 15 | 208887 | Negative |  |  |  |  |  |  |  |
|  | 115 | 95104287460 | Positive |  |  |  |  |  |  |  |
| 1239815 | 123 | 9 | Positive | 20,42 | 29,81 | 30 | 31,92 | Positive | Positive | Included |
|  | 98 | 95107139245 | Positive |  |  |  |  |  |  |  |
|  | 15 | 208887 | Negative |  |  |  |  |  |  |  |
| 14512814 | 145 | 19 | Positive | 17,64 | 29,76 | 30,07 | 30,77 | Positive | Positive | Included |
|  | 128 | 11 | Positive |  |  |  |  |  |  |  |
|  | 14 | 208920 | Negative |  |  |  |  |  |  |  |
| 16511114 | 165 | 27 | Positive | 18,81 | 31,03 | 32 | 31,68 | Positive | Positive | Included |
|  | 111 | 5 | Positive |  |  |  |  |  |  |  |
|  | 14 | 208920 | Negative |  |  |  |  |  |  |  |
| 9714614 | 97 | 95107019263 | Positive | 13,81 | 34,76 | 35,96 | 29,21 | Positive | Positive | Included |
|  | 146 | 20 | Positive |  |  |  |  |  |  |  |
|  | 14 | 208920 | Negative |  |  |  |  |  |  |  |
| 10510914 | 105 | 4 | Positive | 13,21 | 33,29 | 33,24 | 29,24 | Positive | Positive | Included |
|  | 109 | 95107089157 | Positive |  |  |  |  |  |  |  |
|  | 14 | 208920 | Negative |  |  |  |  |  |  |  |
| 9112913 | 91 | 95107019050 | Positive | 13,12 | 35,92 | 37,36 | 30,22 | Positive | Positive | Included |
|  | 129 | 12 | Positive |  |  |  |  |  |  |  |
|  | 13 | 208895 | Negative |  |  |  |  |  |  |  |
| 101223 | 122 | 8 | Positive | 15,77 | 32,75 | 33,7 | 26,6 | Positive | Positive | Included |
|  | 232 | 209105 | Negative |  |  |  |  |  |  |  |
| 106223 | 122 | 8 | Positive | 13,08 | 29,65 | 30,83 | 29,37 | Positive | Positive | Included |
|  | 231 | 209071 | Negative |  |  |  |  |  |  |  |
| 901929 | 99 | 3 | Positive | 15,62 | 31,84 | 33,24 | 30,28 | Positive | Positive | Included |
|  | 229 | 209104 | Negative |  |  |  |  |  |  |  |
| 906927 | 99 | 3 | Positive | 13,37 | 31,82 | 32,85 | 32,07 | Positive | Positive | Included |
|  | 227 | 209093 | Negative |  |  |  |  |  |  |  |
| 101402 | 140 | 17 | Positive | 16,95 | 33,34 | 34 | 31,46 | Positive | Positive | Included |
|  | 226 | 209098 | Negative |  |  |  |  |  |  |  |
| 106401 | 140 | 17 | Positive | 15,10 | 33,87 | 35,17 | 29,91 | Positive | Positive | Included |
|  | 215 | 208825 | Negative |  |  |  |  |  |  |  |

**Table S4: Results obtained by the blind analysis of the 60 samples and their inclusion or exclusion included from the trial.**

| **ID blind trial** | **Sample ID** | **Ct Roche Kit** | **Ct Abbott Kit** | **Ct Hyris Kit Mix 1** | **Ct Hyris Kit Mix 2** | **Ct Hyris Kit RP** | **Roche Kit o Abbott Kit result** | **Hyris Kit result** | **Inclusion in the trial** |
| --- | --- | --- | --- | --- | --- | --- | --- | --- | --- |
| 1 | 208786 |  | N/A | N/A | N/A | 31,27 | Negative | Negative | Included |
| 2 | 5174707 | 16,11 |  | 18,23 | 18,98 | 27,79 | Positive | Positive | Included |
| 3 | 5147607 | 27,71 |  | 30,65 | 32,04 | 29,62 | Positive | Positive | Included |
| 4 | 208829 |  | N/A | N/A | N/A | 31,41 | Negative | Negative | Included |
| 5 | 208788 |  | N/A | N/A | N/A | 31,78 | Negative | Negative | Included |
| 6 | 5193507 | 32,71 |  | 36,18 | 38,51 | 27,48 | Positive | Positive | Included |
| 7 | 208842 |  | N/A | N/A | N/A | 33,22 | Negative | Negative | Included |
| 8 | 500407 | 22,08 |  | 26,62 | 27,72 | 30,62 | Positive | Positive | Included |
| 9 | 208711 |  | N/A | N/A | N/A | 30,72 | Negative | Negative | Included |
| 10 | 208873 |  | N/A | N/A | N/A | 28,43 | Negative | Negative | Included |
| 11 | 5278607 | 32,47 |  | 35,54 | 36,94 | 26,87 | Positive | Positive | Included |
| 12 | 469207 | 18,98 |  | 22,8 | 23,68 | 30,35 | Positive | Positive | Included |
| 13 | 00308 | 24,46 |  | 30,25 | 31,25 | 27,17 | Positive | Positive | Included |
| 14 | 208820 |  | N/A | N/A | N/A | 32,52 | Negative | Negative | Included |
| 15 | 208813 |  | N/A | N/A | N/A | 28,01 | Negative | Negative | Included |
| 16 | 5278707 | 34,25 |  | 37,22 | 36,92 | 28,28 | Positive | Positive | Excluded |
| 17 | 94508 | 32,94 |  | 35,08 | 36,93 | 30,07 | Positive | Positive | Included |
| 18 | 881008 | 30,36 |  | 31,99 | 33,07 | 28,38 | Positive | Positive | Included |
| 19 | 208808 |  | N/A | N/A | N/A | 29,62 | Negative | Negative | Included |
| 20 | 208821 |  | N/A | N/A | N/A | 31,59 | Negative | Negative | Included |
| 21 | 95901 | N/A |  | N/A | N/A | N/A | Negative | Inconclusive | Excluded |
| 22 | 208807 |  | N/A | N/A | N/A | 31,57 | Negative | Negative | Included |
| 23 | 5003608 | 35,39 |  | 34,6 | 36,26 | 28,98 | Negative | Positive | Excluded |
| 24 | 208833 |  | N/A | N/A | N/A | 29,22 | Negative | Negative | Included |
| 25 | 208803 |  | N/A | N/A | N/A | 30,59 | Negative | Negative | Included |
| 26 | 640407 | 38,98 |  | N/A | N/A | 32,99 | Negative | Negative | Excluded |
| 27 | 5146607 | 36,84 |  | 36,28 | 37,63 | 25,78 | Negative | Positive | Excluded |
| 28 | 14708 | 23,09 |  | 26,1 | 27,26 | 27,9 | Positive | Positive | Included |
| 29 | 208818 |  | N/A | N/A | N/A | 30,35 | Negative | Negative | Included |
| 30 | 876107 | 30,66 |  | N/A | 38,89 | 30,02 | Positive | Inconclusive | Excluded |
| 31 | 208823 |  | N/A | N/A | N/A | 30,06 | Negative | Negative | Included |
| 32 | 208826 |  | N/A | N/A | N/A | 30,24 | Negative | Negative | Included |
| 33 | 208889 |  | N/A | N/A | N/A | 28,07 | Negative | Negative | Included |
| 34 | 208914 |  | N/A | N/A | N/A | 30,22 | Negative | Negative | Included |
| 35 | 122245 |  | 12,55 | 29,55 | 31,34 | 29,05 | Positive | Positive | Included |
| 36 | 122247 |  | 12,68 | 29,1 | 30,57 | 29,66 | Positive | Positive | Included |
| 37 | 208865 |  | N/A | N/A | N/A | 29,98 | Negative | Negative | Included |
| 38 | 9924 |  | 12,55 | 29,93 | 31,27 | 27,46 | Positive | Positive | Included |
| 39 | 208858 |  | N/A | N/A | N/A | 28,61 | Negative | Negative | Included |
| 40 | 9912729 |  | 13,04 | 28,75 | 30,55 | 29,37 | Positive | Positive | Included |
| 41 | 9916418 |  | 14,08 | 29,6 | 30,94 | 29,44 | Positive | Positive | Included |
| 42 | 208870 |  | N/A | N/A | N/A | 26,75 | Negative | Negative | Included |
| 43 | 9912723 |  | 13,86 | 29,25 | 30,31 | 32,29 | Positive | Positive | Included |
| 44 | 208850 |  | N/A | N/A | N/A | 30,48 | Negative | Negative | Included |
| 45 | 208897 |  | N/A | N/A | N/A | 29,67 | Negative | Negative | Included |
| 46 | 14022 |  | 13,6 | 30,33 | 31,65 | 33,58 | Positive | Positive | Included |
| 47 | 122164248 |  | 12,84 | 29,9 | 31,65 | 30,77 | Positive | Positive | Included |
| 48 | 1409816 |  | 15,23 | 30,96 | 32,7 | 28,88 | Positive | Positive | Included |
| 49 | 208893 |  | N/A | N/A | N/A | 29,45 | Negative | Negative | Included |
| 50 | 14021 |  | 19,05 | 30 | 31,14 | 32,1 | Positive | Positive | Included |
| 51 | 122219 |  | 12,62 | 29,45 | 31,72 | 31,25 | Positive | Positive | Included |
| 52 | 208869 |  | N/A | N/A | N/A | 29,46 | Negative | Negative | Included |
| 53 | 14016017 |  | 15,13 | 29,82 | 31,39 | 31,87 | Positive | Positive | Included |
| 54 | 208860 |  | N/A | N/A | N/A | 27,25 | Negative | Negative | Included |
| 55 | 122104249 |  | 12,35 | 29,36 | 31,37 | 29,26 | Positive | Positive | Included |
| 56 | 208667 |  | N/A | N/A | N/A | 29,23 | Negative | Negative | Included |
| 57 | 99212 |  | 12,63 | 28,89 | 30,02 | 32,34 | Positive | Positive | Included |
| 58 | 208896 |  | N/A | N/A | N/A | 32,14 | Negative | Negative | Included |
| 59 | 208905 |  | N/A | N/A | N/A | 29,95 | Negative | Negative | Included |
| 60 | 208876 |  | N/A | N/A | N/A | 30,46 | Negative | Negative | Included |
